# Edible bird's nest improves the premature ovarian failure induced by tripterygium glycosides

**DOI:** 10.1002/fsn3.4119

**Published:** 2024-03-21

**Authors:** Lin Jiang, Xuncai Liu, Fenghong Deng, Yaxin Wang, Qunyan Fan

**Affiliations:** ^1^ School of Pharmaceutical Sciences Sun Yat‐Sen University Guangzhou China; ^2^ Bird's Nest Research Institute, Xiamen Yan Palace Seelong Biotechnology Co., Ltd. Xiamen China

**Keywords:** edible bird's nest, improvement, premature ovarian failure, tripterygium glycosides

## Abstract

Premature ovarian failure (POF) is a common disease in the field of gynecological endocrinology that seriously affects the physical and mental health of patients. Previous studies found that edible bird's nest (EBN) could improve uterine function. These suggested that EBN might also have an ameliorating effect on POF. Therefore, in this study, tripterygium glycosides (TGs) were used to induce POF in rats, and the effect of EBN on the improvement of POF was investigated. After the administration of EBN for 14 days, ovarian index and uterine index, serum hormone levels, apoptosis rate of ovarian granulosa cells, follicle‐stimulating hormone receptor (FSHR) protein expression level, and the histopathological examination of the ovaries were determined. It was found that administration of medium and high EBN dose groups increased the ovarian index and granular layer thickness of rats with POF. Particularly, higher follicle‐stimulating hormone levels and lower corpus luteum content were observed in the high EBN dose group. In addition, there were lower luteinizing hormone levels and fewer atretic follicles but higher progesterone levels in the medium EBN dose group. These results indicated that EBN had preventive and curative effects on POF induced by TGs. Its mechanism of action might be related to the reduction of ovarian granulosa cell apoptosis, regulation of hormones and receptors, and inhibition of follicle closure.

## Graphical abstract

This study investigated the ameliorating effect of EBN on tripterygium glycoside‐induced POF. It was found that EBN could improve ovarian function, and the mechanism of action might be related to the reduction of ovarian granulosa cell apoptosis, regulation of hormones and receptors, and inhibition of follicle closure.

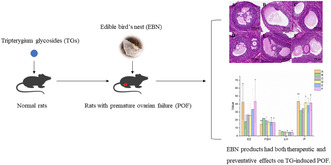

## 1 INTRODUCTION

Primary ovarian failure (POF), another name for premature ovarian insufficiency (POI), is a condition that affects women under 40 years of age and is characterized by ovarian failure symptoms like amenorrhea, infertility, low serum estrogen levels, and elevated gonadotropin levels (Ebrahimi & Asbagh, 2011; Welt, 2008; Wesevich et al., 2020). In the realm of gynecological endocrinology, it is a prevalent illness. Low estrogen and high gonadotropin levels are its defining characteristics, which lead to amenorrhea and infertility and negatively impact women's physical and mental health (Jankowska, 2017; Wesevich et al., 2020). POF had a complex etiology, which contributed to its yearly rise in prevalence and growing medical community interest. Although the exact causes of POF were still unknown, the following theories had been put forth: autoimmune‐related variables, drugs, metabolism, and genes (Jiao et al., 2017; Rudnicka et al., 2018; Torrealday et al., 2017; Woad et al., 2006). In addition, follicle growth and proliferation and ischemia reperfusion injury could also lead to POF (Atilgan et al., 2015; Tuncer et al., 2023). Other studies had found that ion channels might also have an impact on ovarian reserve and became one of the factors affecting POF (Şanlı et al., 2021; Atilgan et al., 2020). Moreover, ovarian tissue damage might be related to oxidative stress. Autophagy, which was induced by oxidative stress, was one of the main pathways of destruction and was essential for cellular homeostasis, as well as maintaining energy production and the synthesis of new macromolecules. Autophagy increased follicular atresia, which led to decreased ovarian reserve (Delkhosh et al., 2019; Majdi Seghinsara et al., 2019). The treatment of POF in modern medicine was not limited to restoring, preserving, and replacing ovarian function, but so far there was no definite and effective way to restore ovarian function. Western medicine relied on hormone replacement therapy, which was highly effective (Vujovic et al., 2010). However, chronic hormone replacement medication use might result in adverse effects like heart disease, thrombosis, breast cancer, and endometrial cancer in women (Dragojevic‐Dikic et al., 2009). Thus, the search for natural materials for POF rescue was essential.

Tripterygium glycosides (TGs) were frequently used to treat arthritis. After using the medication, some women developed amenorrhea and menstrual problems. Therefore, TGs became one of the medical factors in POF (Han et al., 2012). It had been discovered in clinical settings that TGs caused ovarian hypofunction and failure, which decreased the amount of estrogen released by the ovaries. The pituitary gland then received these alterations, which led to an overabundance of gonadotropin output, including luteinizing hormone (LH), follicle‐stimulating hormone (FSH), and even amenorrhea (Ai et al., 2018; Su et al., 2014). Therefore, TGs might be utilized to create an animal POF model.

Edible bird's nest (EBN), a kind of natural tonic, has high nutritional value. The main components of EBN were carbohydrate (25.6%–27.3%), protein (62.0%–63.0%), sialic acid (~10.0%), and ash (~2.1%) (Hun Lee et al., 2020; Marcone, 2005; Quek et al., 2018). It was reported that EBN could upregulate the expression of genes of epidermal growth factor, vascular endothelial growth factor, proliferating cell nuclear antigen, progesterone receptors, and estrogen receptors in the uterus (Albishtue et al., 2018; Albishtue, Yimer, Zakaria, Haron, Babji, Abubakar, & Almhanawi, 2019; Albishtue, Yimer, Zakaria, Haron, Babji, Abubakar, Baiee, et al., 2019; Roh et al., 2012). In addition, EBN could increase serum estrogen levels in de‐ovulatory rats (Hou et al., 2017). These studies suggested that consumption of EBN might be an effective adjuvant treatment for POF.

Therefore, in this study, a rat model of POF was established by administering the rats intragastrically with TGs, and the effect of EBN on the improvement of TG‐induced POF was investigated. This study will be conducive to enriching the pharmacological effects of EBN and developing related products to improve POF.

## 2 MATERIALS AND METHODS

### 2.1 Materials

EBN was provided by Xiamen Yan Palace Seelong Biotechnology Co., Ltd., which was identified as genuine by Lin Jiang, a researcher in Chinese herbal medicine at Sun Yat‐Sen University. The TG tablets were obtained from Yuanda Pharmaceutical Huangshi Feiyun Pharmaceutical Co., Ltd. The combined estrogen tablets were from Xinjiang Xinziyuan Biopharmaceutical Co., Ltd.

### 2.2 Animal experiments and ethics approval

Female SD rats of 8–9 weeks in age were provided by Guangdong Medical Laboratory Animal Center (SCXK (Guangdong) 2022–0002, Certificate No.: 44007200111747). The rats were kept in sterile cages in the Laboratory Animal Center North Campus of Sun Yat‐sen University (License No.: SYXK (Guangdong) 2022–0081), which was controlled with a temperature of 20–25°C, a relative humidity of 40%–70% and a 12 h light/12 h dark cycle.

**TABLE 1:** The drug application doses and days and the rat groups.

Rat groups	Drug application dose	Drug application days
Model control group	Equal volume of distilled water	14 days
Low EBN group	0.45 g EBN/kg/day	
Medium EBN group	0.675 g EBN/kg/day	
High EBN group	0.9 g EBN/kg/day	
Positive control combined with estrogen	0.1 mg combined estrogen tablet/kg/day	

### 2.3 POF model establishment

All rats had been acclimatized for 7 days. The SPF‐grade SD female rats were subjected to exfoliative cytoscopy of the vagina for 10 consecutive days. Then, the rats with a normal estrous cycle were set as animal subjects, and the rats with an abnormal estrous cycle were left out of the study. 10 rats were chosen randomly as the normal control (A group), while others were administered intragastrically with TGs at 75 mg/kg/day for 14 days. After that, exfoliative cytoscopy of the vagina was performed again, and the estrous cycle was observed. Disordered estrous cycles indicated that the POF model was established successfully, and rats with unsuccessful modeling were left out of the study. Fifty successfully modeled rats were randomly grouped and then continued to be administered 75 mg TGs/kg once daily for 14 days to ensure the success of the model.

### 2.4 Grouping and drug administration

The EBN was soaked for 4 h and then stewed at 100°C for 30 min. The stewed EBN was stored at −20°C and melted in a hot bath before use.

The successfully modeled rats were randomly divided into 5 groups (Table 1): Model control group (B group, distilled water), low EBN dose group (C group, 0.45 g EBN/kg/day), medium EBN dose group (D group, 0.675 g EBN/kg/day), high EBN dose group (E group, 0.9 g EBN/kg/day), and positive control combined with estrogen group (F group, 0.1 mg combined Estrogen Tablet/kg/day). After the rats were fed the same volume of different drugs, blood samples were collected, and serum was separated for use 14 days later. Then the rats were sacrificed and dissected, and the organs were used for index determination and histomorphological observation.

### 2.5 General status and weight observation

Weighing and general status observations were performed weekly before and after the administration of the drug.

### 2.6 Ovarian index and uterine index

The ovary and uterus were taken and weighed, and then the organ index was calculated according to the following formula: Organ index = organ weight (mg)/body weight (g).

### 2.7 Serum hormone levels

The levels of FSH, LH, estradiol (E2), and progesterone (P) in each group of rats were measured by the chemiluminescence method.

### 2.8 Apoptosis rate of ovarian granulosa cells

After the rats were dissected, the left ovarian tissue was fixed for paraffin sectioning, and the apoptotic rate of ovarian granulosa cells was determined by the terminal deoxynucleotidyl transferase‐mediated end‐labeling technique (TUNEL method).

### 2.9 Follicle stimulating hormone receptor (FSHR) protein expression level

After the rats were dissected, the left ovarian tissues were fixed for paraffin sectioning, and the FSHR protein expression level was detected by immunohistochemistry.

### 2.10 Histopathological examination of the ovaries

The left ovarian tissues were fixed, embedded, paraffin‐sectioned, and HE‐stained. Then the changes in the ovarian tissues of rats were observed under a light microscope, including the number of primordial follicles, secondary follicles, sinus follicles, atretic follicles, and corpus luteum, and the thickness of the granulosa cell layer of sinus follicles.

### 2.11 Statistical analysis

There were 10 samples in each group, and each experiment was repeated 3 times per sample. The final data were presented as the means ± standard deviation. SPSS software (Version 26.0, IBM Corp, Armonk, NY, USA) was utilized for one‐way analysis of variance (ANOVA), and the Tukey's test was used to analyze the differences among the mean values. Compared with the model group, if *p* < .05, the difference between the two groups was considered significant, which was indicated by *; if *p* < .01, the difference between the two groups was considered highly significant, which was indicated by **. In addition, the Origin 2022 software (OriginLab, USA) was used for graphing, and the error bar on the bar chart represents the standard deviation.

## 3 RESULTS

### 3.1 Body weight and general status of rats with POF

**FIGURE 1:**
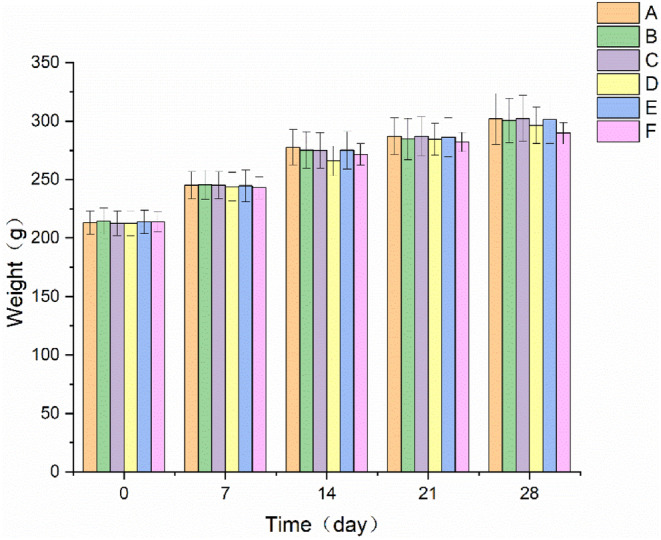
Effect of EBN on body weight of rats with POF (mean ± SD, *n* = 10). A. Normal control group, B. Model control group, C. Low EBN dose group, D. Medium dose group, E. High EBN dose group, F. Positive control group.

During the experimental period, the rats in all groups were in good mental condition. The rats moved freely, breathed evenly, did not show any significant abnormalities in food intake or feces, and had no abnormal secretions in their mouths and noses. As shown in Figure 1, the body weight of the rats in each group increased normally during each period of administration, and there were no significant differences in the body weight of the rats in each sample group compared with the normal control group.

### 3.2 EBN shortened the estrous cycle of rats with POF

**FIGURE 2:**
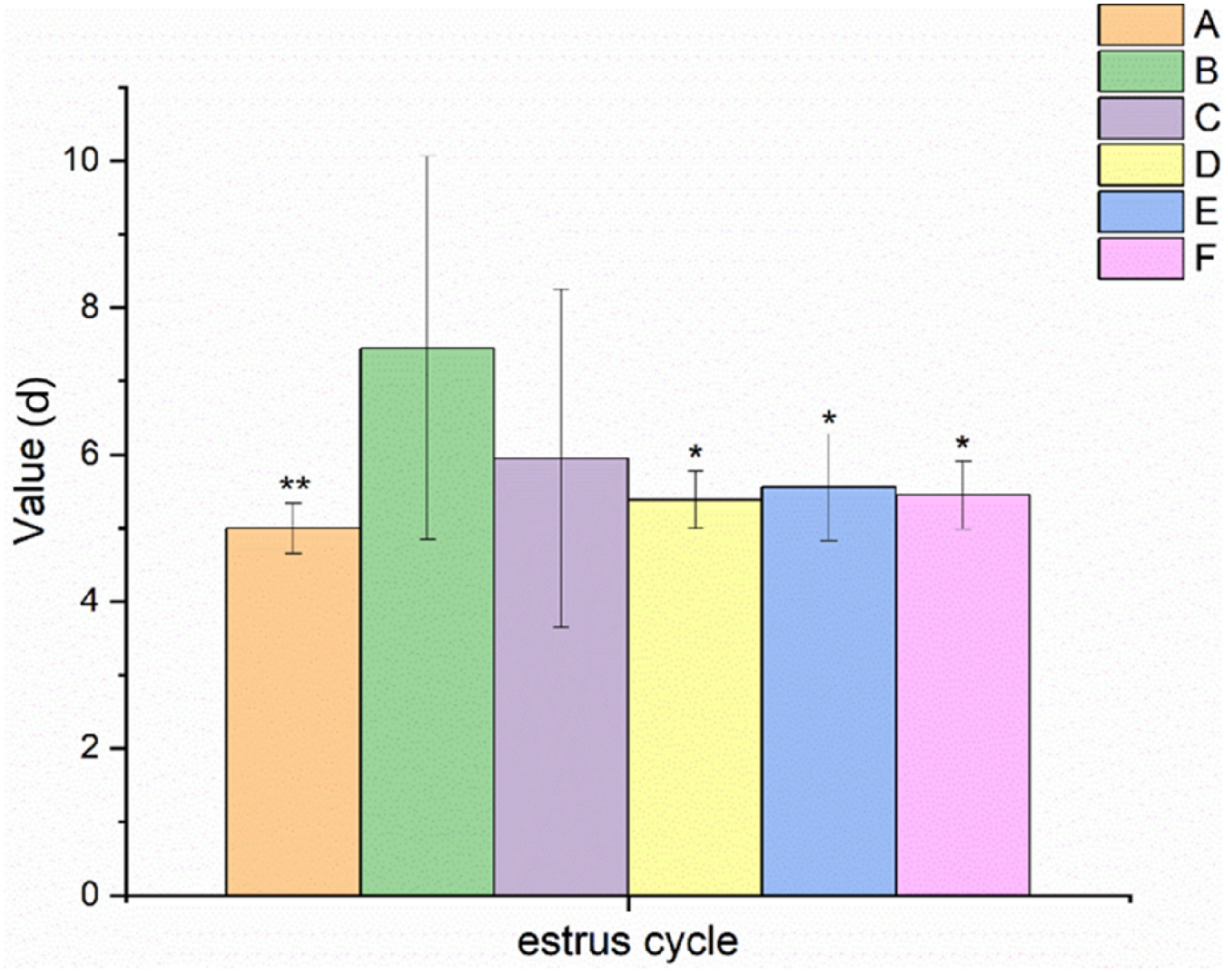
Effect of EBN on estrous cycle in rats with POF (mean ± SD, *n* = 10). A. Normal control group, B. Model control group, C. Low EBN dose group, D. Medium dose group, E. High EBN dose group, F. Positive control group. Compared with model group, if *p* < 0.05, the difference between the two groups was considered significant, which was indicated by *; If *p* < 0.01, the difference between the two groups was considered highly significant, which was indicated by **.

As shown in Figure 2, the estrous cycle of the model group was significantly prolonged (*p* < .01) after 28 days of continuous administration of TGs compared with that of the normal control group. It was suggested that the rat model of POF was successfully established. Compared with the estrous cycle of the model control group, that of rats with POF was significantly shorter in the medium and high EBN dose groups (*p* < .05).

### 3.3 EBN increased the ovarian index of rats with POF

**FIGURE 3:**
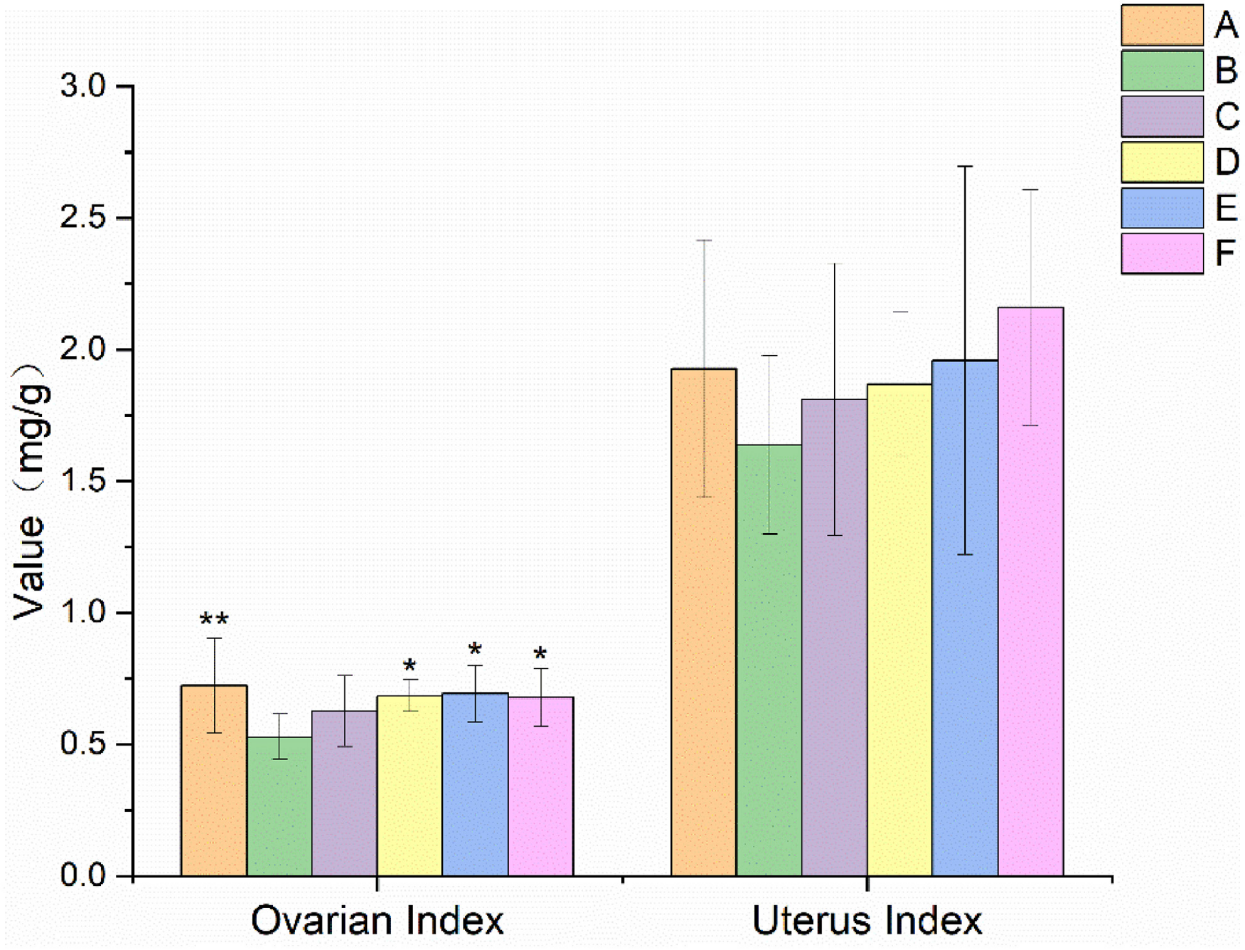
Effect of EBN on ovarian index and uterine index in rats with POF (mean ± SD, *n* = 10). A. Normal control group, B. Model control group, C. Low EBN dose group, D. Medium dose group, E. High EBN dose group, F. Positive control group. Compared with model group, if *p* < 0.05, the difference between the two groups was considered significant, which was indicated by *; If *p* < 0.01, the difference between the two groups was considered highly significant, which was indicated by **.

As shown in Figure 3, the ovarian index of rats in the model group was significantly lower (*p* < .01) than that of rats in the normal control group. However, there was no statistical difference in the uterine index. Compared with the ovarian index of rats in the model control group, that of rats in the middle and high EBN dose groups increased significantly (*p* < .05). However, no statistical difference was found in the uterine index of rats in each EBN dose group. The ovarian index of rats in the positive control combined with the estrogen group increased significantly (*p* < .05), and the uterine index tended to increase.

### 3.4 EBN helped to restore the serum hormone levels of rats with POF

**FIGURE 4:**
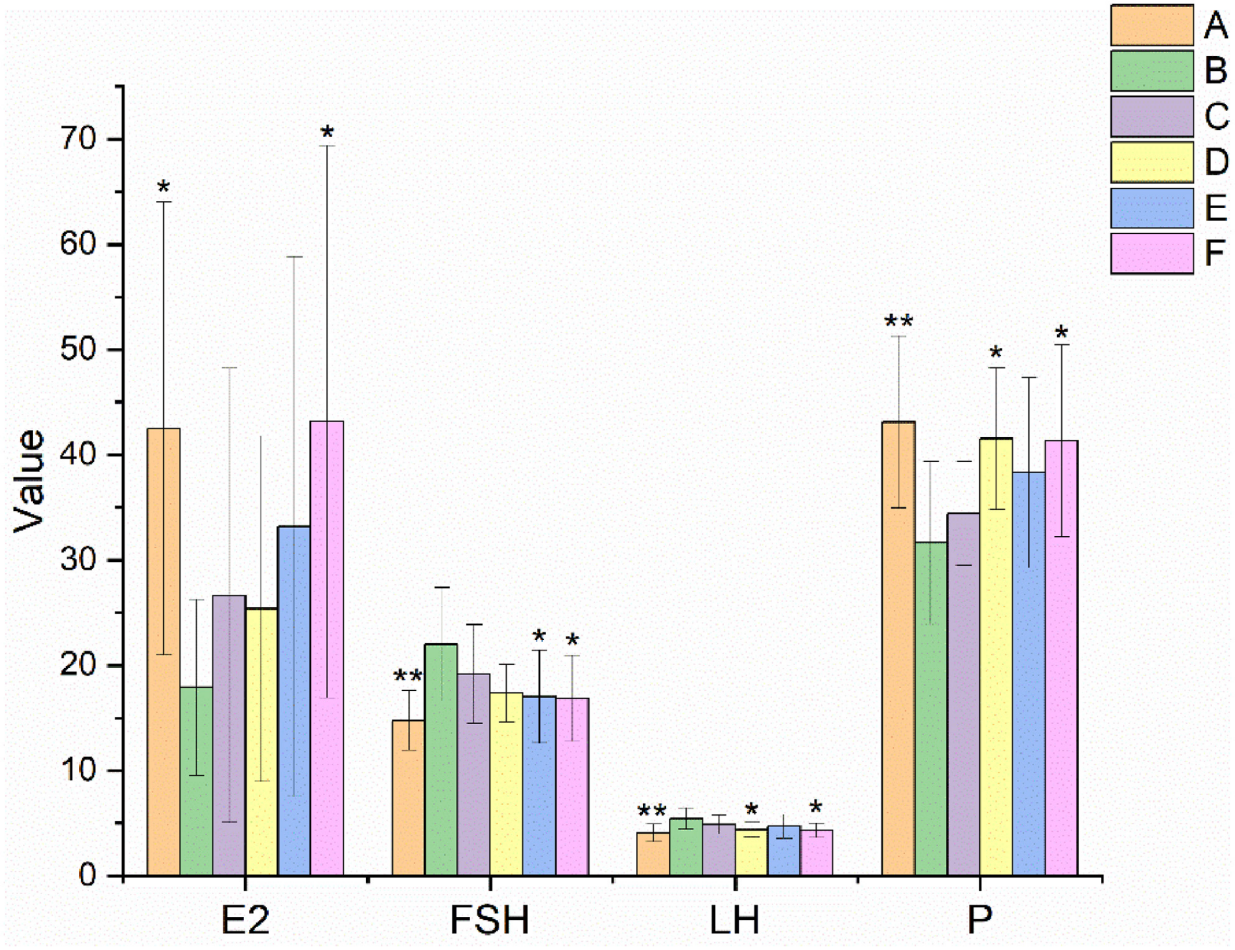
Effect of EBN on serum hormone levels of rats with POF (mean ± SD, *n* = 10). A. Normal control group, B. Model control group, C. Low EBN dose group, D. Medium dose group, E. High EBN dose group, F. Positive control group. Compared with model group, if *p* < 0.05, the difference between the two groups was considered significant, which was indicated by *; If *p* < 0.01, the difference between the two groups was considered highly significant, which was indicated by **.

The serum hormones of rats in each group are shown in Figure 4. The E2 and *p* levels were significantly decreased (*p* < .05 or *p* < .01) and the FSH and LH levels were significantly increased (*p* < .01) in the model group compared with the normal control group, respectively. LH levels were significantly decreased (*p* < .05) and P levels were significantly increased (*p* < .05) in the medium EBN dose group compared with the model control group, respectively. Differently, FSH levels were significantly decreased (*p* < .05) in the high EBN dose group compared with the model control group.

### 3.5 EBN helped to decrease the apoptosis rate of ovarian granulosa cells in rats with POF

**FIGURE 5:**
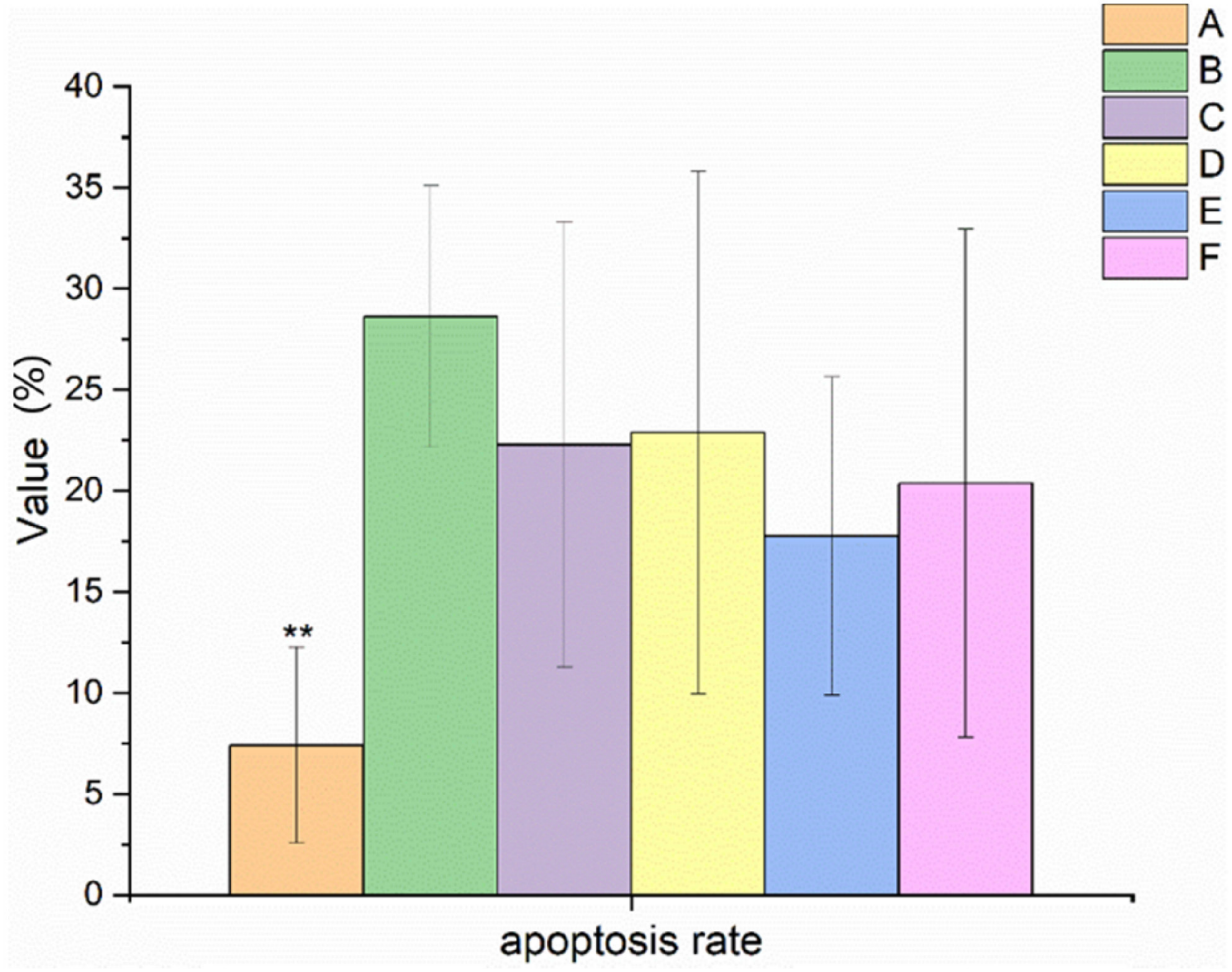
Effect of EBN on the apoptosis rate of granulosa cells in rats with POF (mean ± SD, *n* = 10). A. Normal control group, B. Model control group, C. Low EBN dose group, D. Medium dose group, E. High EBN dose group, F. Positive control group. Compared with model group, if *p* < 0.05, the difference between the two groups was considered significant, which was indicated by *; If *p* < 0.01, the difference between the two groups was considered highly significant, which was indicated by **.

The result of ovarian granulosa cell apoptosis in rats with POF is shown in Figure 5. It was found that the apoptosis rate of ovarian granulosa cells in the model control group increased significantly (*p* < .01) after 28 days of continuous administration of TGs. Compared with the model control group, the apoptosis rate of granulosa cells in rats in the high EBN dose group tended to decrease.

### 3.6 Effect of EBN on follicles at all levels of ovaries in rats with POF

**FIGURE 6:**
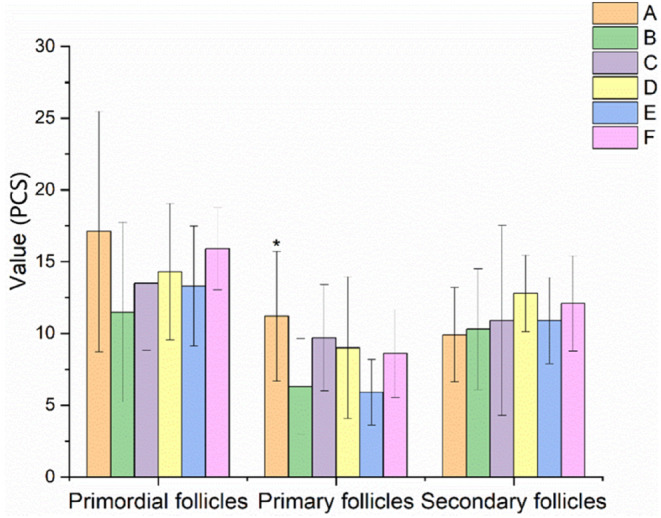
Effects of EBN on follicles at all levels in rats with POF (mean ± SD, *n* = 10). A. Normal control group, B. Model control group, C. Low EBN dose group, D. Medium dose group, E. High EBN dose group, F. Positive control group. Compared with model group, if *p* < 0.05, the difference between the two groups was considered significant, which was indicated by *; If *p* < 0.01, the difference between the two groups was considered highly significant, which was indicated by **.

**FIGURE 7:**
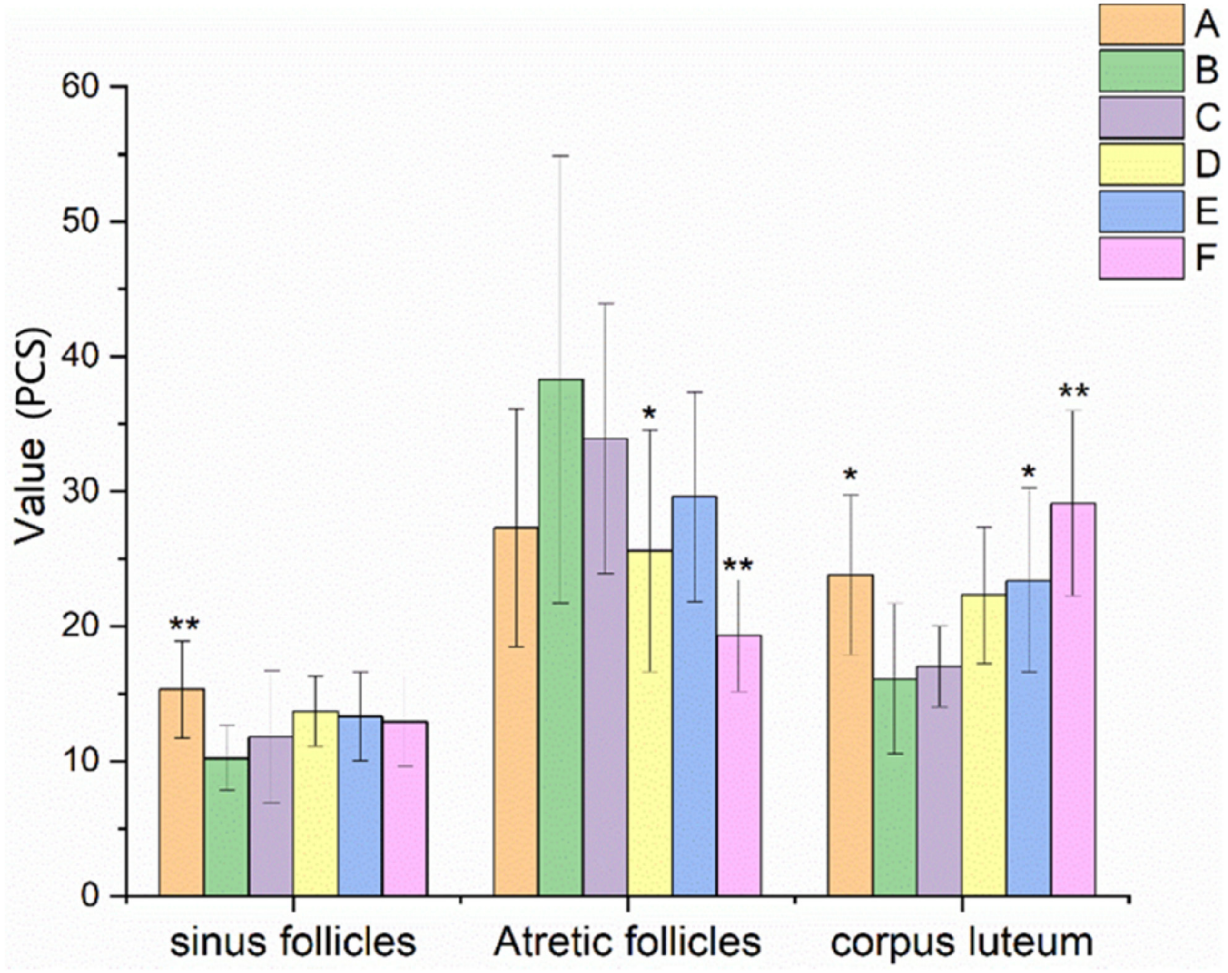
Effects of EBN on follicles at all groups of rats with POF (mean ± SD, *n* = 10). A. Normal control group, B. Model control group, C. Low EBN dose group, D. Medium dose group, E. High EBN dose group, F. Positive control group. Compared with model group, if *p* < 0.05, the difference between the two groups was considered significant, which was indicated by *; If *p* < 0.01, the difference between the two groups was considered highly significant, which was indicated by **.

The results of follicles at all levels in rats with POF are shown in Figures 6 and 7. There was a trend of decrease in the level of primordial follicles, a significant decrease in the number of primary follicles, sinus follicles and atretic follicles (*p* < .05 or *p* < .01), while there was an increase in the number of corpus luteum in the model control group after 28 days of continuous administration of TGs. And no statistical difference was found in secondary follicles between the control and experiment groups.

Compared with the model control group, the number of sinus follicles in the medium and high EBN dose groups and the positive control combined with estrogen tended to increase. However, the number of atretic follicles in the medium EBN dose group and the positive control group significantly decreased (*p* < .05 or *p* < .01). In addition, the number of corpus luteum in the high EBN dose group and the positive control group significantly increased (*p* < .05 or *p* < .01). Moreover, there was an increasing trend in the number of luteal bodies in medium‐dose EBN group.

### 3.7 EBN increased the thickness of the ovarian granular layer

**FIGURE 8:**
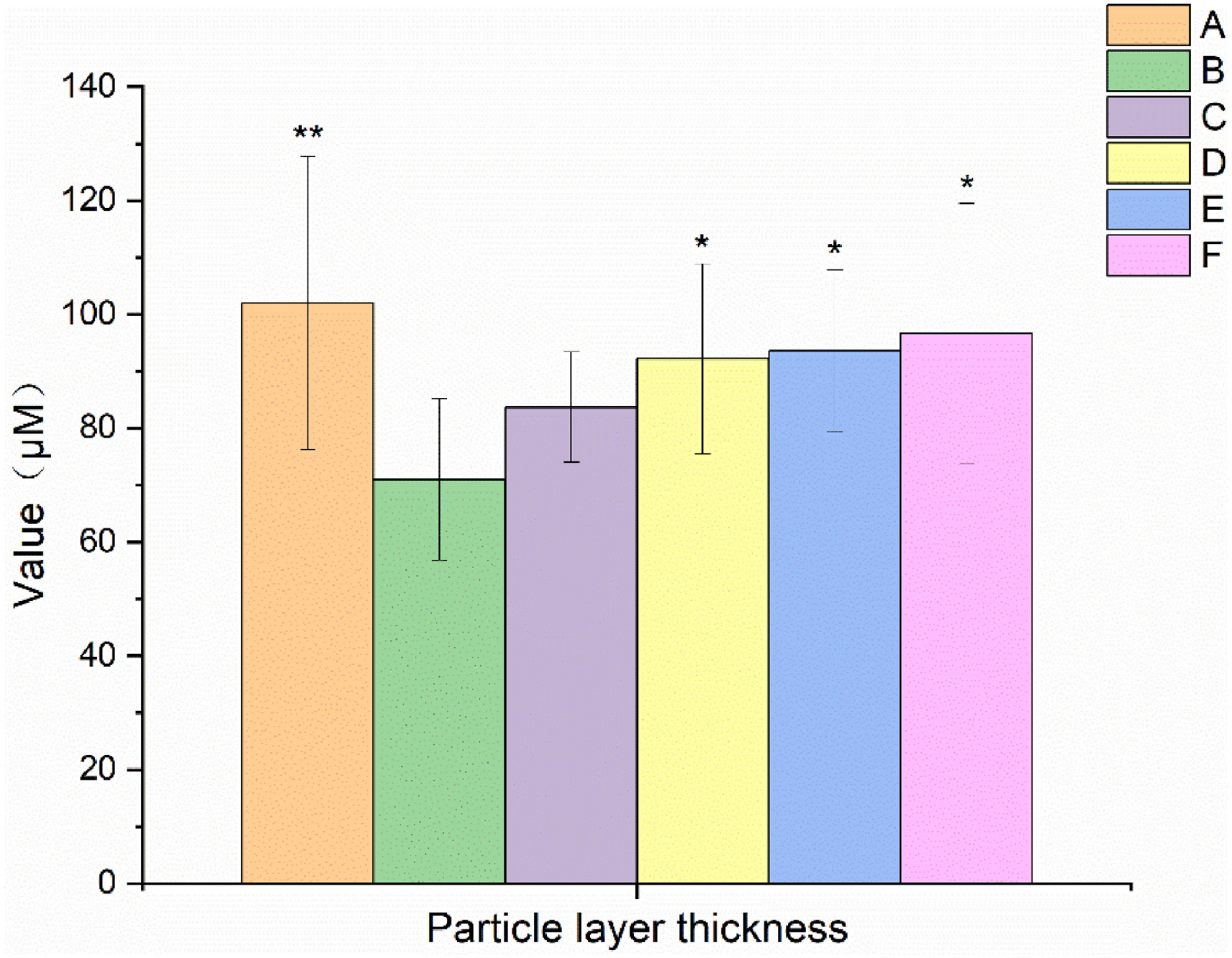
Effects of EBN on the thickness of ovarian granular layer in rats with POF (mean ± SD, *n* = 10). A. Normal control group, B. Model control group, C. Low EBN dose group, D. Medium dose group, E. High EBN dose group, F. Positive control group. Compared with model group, if *p* < 0.05, the difference between the two groups was considered significant, which was indicated by *; If *p* < 0.01, the difference between the two groups was considered highly significant, which was indicated by **.

**FIGURE 9:**
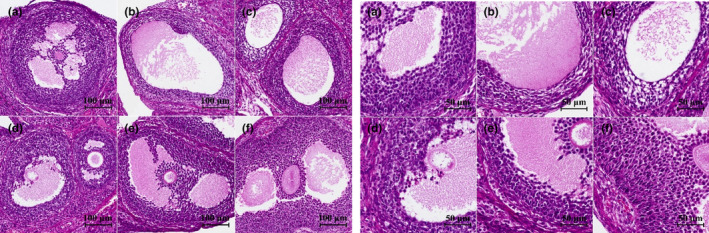
Representative diagram of the effect of EBN on the histopathology of ovaries of rats with POF (HE staining). (a) Normal control group, (b) Model control group, (c) Low EBN dose group, (d) Medium EBN dose group, (e) High EBN dose group, (f) Positive control group.

The results of ovarian granular layer thickness in rats with POF are shown in Figures 8 and 9. It was found that the thickness of the ovarian granular layer in the model control group was significantly reduced (*p* < .01) after 28 days of continuous administration of TGs. Compared with the model control group, the thickness of the ovarian granular layer was significantly increased in the middle and high EBN doses and the positive control combined with the estrogen group (*p* < .05).

### 3.8 EBN helped to downregulate the expression of FSHR protein in rats with POF

**FIGURE 10:**
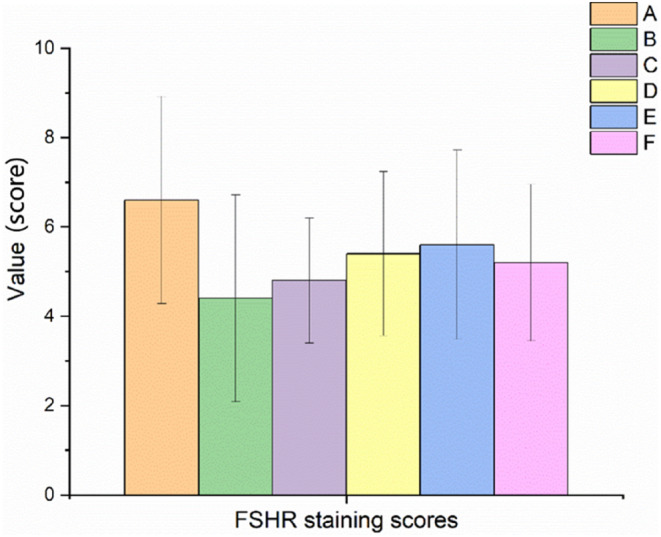
Effect of EBN on the immunohistochemical staining score of ovarian FSHR in rats with POF (Mean ± SD, *n* = 10). A. Normal control group, B. Model control group, C. Low EBN dose group, D. Medium dose group, E. High EBN dose group, F. Positive control group.

**FIGURE 11:**
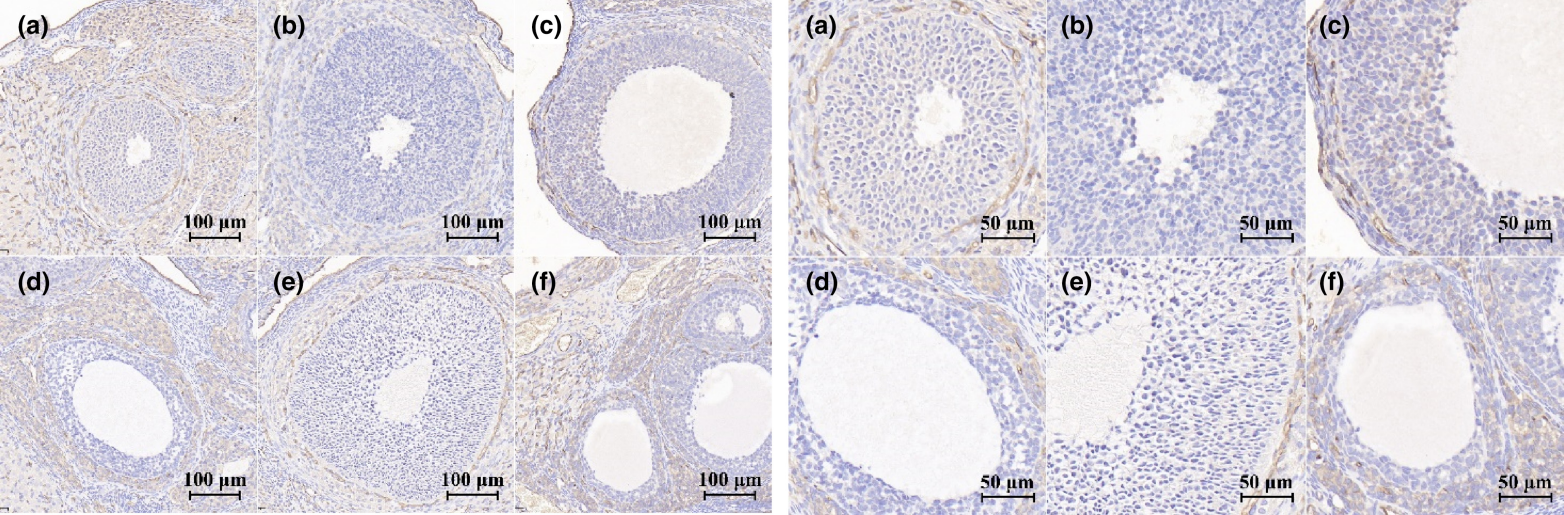
Representative plots of the effects of EBN on ovarian FSHR protein expression in rats with POF (immunohistochemical staining). (a) Normal control group, (b) Model control group, (c) Low EBN dose group, (d) Medium dose group, (e) High EBN dose group, (f) Positive control group.

The results of ovarian FSHR protein expression in rats with POF are shown in Figures 10 and 11. It was demonstrated that the expression of FSHR protein was downregulated in the model control group after 28 days of continuous administration of TGs. Compared with the model control group, there was an upregulation trend in the expression of FSHR protein in each EBN‐treated group and positive control group, but there was no statistical difference.

## 4 DISCUSSION

In this study, the rats in the model group showed a prolonged estrous cycle. The ovarian and uterine index, E2 and P levels, particle layer thickness, and FSHR protein expression were decreased. FSH and LH levels, the apoptosis rate of granulosa cells, and the number of atretic follicles increased. After the rats were treated with different doses of EBN, the ovarian and uterine index, E2 and P levels, particle layer thickness, and FSHR protein expression were all increased. The estrous cycle, FSH and LH levels, apoptosis rate of granulosa cells, and number of atretic follicles were all decreased.

In the clinical management of patients with POF, sex hormones such as E2, FSH, LH, and P were used as indicators for POF (Qiu et al., 2022). Among them, E2 and FSH were the direct diagnostic indicators, and LH and P were the indirect indicators. These hormones were regulated by the hypothalamic–pituitary–ovarian (HPO) axis. Specifically, gonadotropin‐releasing hormone (GnRH), which was secreted by the hypothalamus, was directly regulated by the gonadotropin‐secreting function of the pituitary, which in turn regulated the production of FSH and LH. FSH was a kind of glycoprotein sex hormone that was synthesized and secreted by the basophils of the anterior pituitary gland. It had the functions of promoting follicle growth and development, granulosa cell proliferation and differentiation, etc. It could cooperate with LH to promote ovulation and luteinization, promote the normal secretion of estrogen and progesterone, and maintain the cycle law of menstrual formation. The production of FSH and LH was not only regulated by GnRH in the hypothalamus but also by positive and negative feedback regulation of E2, which affected the production of E2, P, and other steroid hormones in the regulated pathway (Mikhael et al., 2019). The dysfunction of the HPO axis caused abnormal hormone secretion in the body, which affected the growth and development of follicles and ovulation function and ultimately affected the reserve function and fertility of the ovary.

The ovary's corpus luteum and follicular cells were the primary sources of E2, which directly impacted ovarian activity. The female reproductive endocrine system's stability and upkeep, as well as the overall health of the organism, depended heavily on the amount of E2. It had been shown that when ovarian tissue was treated with estrogen in vitro, the growth of the tissue was accelerated. The development, maturity, and ovulation of follicles depended heavily on estrogen. Increased blood flow to the uterus, protection of ovarian function, endometrial repair and proliferation, and follicle growth and development in the ovary were all potential benefits of estrogen (Britt et al., 2004). Consequently, E2 might be considered a direct measure of ovarian reserve capacity, and a marked decline in E2 concentration indicated a reduction in the ovaries' capacity to release estrogen. Granulosa cells in the ovarian corpus luteum generated P, which was a natural progestogen secreted by the ovaries. P enhanced the amount of glycogen and endometrial cell volume, and the secretory glands released mucus into the secretion that contained glycogen, guaranteeing the success of fertilization.

E2 and P levels in serum were important indicators to evaluate ovarian function. The values of E2 and P in the model control group (B) were significantly lower than those in the normal control group (A), and the E2 and P levels in the EBN treatment groups (C, D, and E) all tended to increase compared to those in the model control group (B). Particularly, the P level in the medium EBN dose group (D) was significantly higher than that in the model control group (B) (*p* < .05). These results indicated that ingestion of EBN increased E2 and P levels and restored the impaired ovarian function caused by TGs.

The anterior pituitary gland released the gonadotropin known as FSH. The main hormone that promoted follicular growth was FSH. The diagnosis of ovarian hypofunction and infertility, as well as the prediction of ovulation, depended on the levels of FSH and LH in the blood. FSH and LH together stimulated follicular maturation and ovulation, serving as markers of the capacity of the ovarian reserve. Increased LH production prompted the ovaries to produce excess androgens. Excessive androgen had adverse effects on the occurrence, development, and maturation of follicles. Androgen entered the granulosa cells of the preantral follicle and bound to the androgen receptor, thereby inducing granulosa cell apoptosis and enhancing the responsiveness of granulosa cells to FSH. This inhibited follicular development, promoted follicular atresia, and reduced the ability of the ovary to synthesize estrogen, which led to HPO axis dysfunction (Ebrahimi et al., 2023). In addition, excess androgens also suppressed the negative feedback effect of estrogen on hypothalamic GnRH pulse generation, resulting in a rapid LH pulse frequency that stimulates premature luteinization of granulosa cells, thereby inhibiting antral folliculogenesis, development, and ovulation. Clinically, increased levels of FSH and LH in women of reproductive age were suggested to be indicative of ovarian dysgenesis, which included primary amenorrhea, ovarian insufficiency, and other disorders (Qiu et al., 2022).

The FSH and LH levels in the EBN treatment groups (C, D, and E) all tended to decline in comparison to those in the model control group (B), while the FSH and LH levels in the model control group (B) were considerably higher than those in the normal control group (A). Furthermore, there was a significant difference (*p* < .05) in the FSH level in the high EBN dose group (E) and the LH level in the medium EBN dose group (D) compared to the model control group (B). It was shown that rats' ovarian function was hampered by TGs. These negative effects subsided after EBN was administered. That is, EBN might improve the POF brought on by TGs through the HPO axis. Similarly, previous studies had found that a period of low dose of clove intake could reduce the LH/FSH ratio and improve ovarian damage (Soltani et al., 2023).

The quantity and quality of follicles in the ovary dictated ovarian function and were related to fertility (Lunenfeld et al., 1975). The follicles' gradual loss of sensitivity to FSH was the first indication of ovarian failure. Early on, high FSH overstimulated the follicles, leading to an overabundance of E2 production. The progressive cessation of follicular growth and development resulted in a decrease in E2 levels and an increase in FSH. Poor ovarian reserve and secretory function were the result of these indicator changes (Lenestour et al., 1993). Follicle count was thus a proxy for the function of ovarian reserve.

The fertility of women of childbearing age was impacted by POF. Women with POF differed from menopausal women in that they still maintained reserves in their ovaries. Thus, it was critical to comprehend how POF patients' ovarian function was preserved or restored. Because of their extreme reproductive toxicity, TGs were frequently employed in experimental POF modeling. Rats displayed disruptions in the estrous cycle, increased FSH and LH levels, and decreased E2 levels during the modeling period. Furthermore, structural and atrophic abnormalities of the ovarian tissue were noted. In addition, there were anomalies in the oocyte and zona pellucida morphology, a decrease in follicles at all levels, and an increase in atretic follicles (Su et al., 2014; Welt, 2008). It took the rats 30 days after the modeling was stopped for their hormone levels and ovarian shape to return to normal, indicating that the model had good stability.

Atresia, fast depletion, and low primary follicular reserve were the main causes of POF. The number of primordial follicles, primary follicles, secondary follicles, and sinus follicles in the EBN‐treated groups (C, D, and E) all tended to increase compared to those in the model control group (B). Moreover, the rate of granulosa cell apoptosis in EBN‐treated groups tended to decrease compared to that of the model control group (B). Specifically, there were substantially fewer atretic follicles in the medium EBN dose group (D) than in the model control group (B) (*p* < .05). In addition, there was a significant difference (*p* < .05) in the number of corpus luteum between the high EBN dose group (E) and the model control group (B). Furthermore, the granulosa layer thickness in the medium and high EBN dose groups (D and E) was significantly higher than that in the model control group (B) (*p* < .05). It was indicated that EBN was conducive to the improvement of POF.

In this study, the improvement of POF by ingestion of EBN was explored using the estrous cycle, hormone levels, ovarian index, uterine index, apoptosis rate of granulosa cells, number of follicles, particle layer thickness, and FSHR protein expression as indicators. EBN products enhanced the ovarian index and P level and considerably shortened the estrous cycle under the current experimental settings. Furthermore, after EBN treatment, the ovary's granular layer thickness and corpus luteum count both increased. There was a notable decrease in both the number of atretic follicles and the levels of FSH and LH. Additionally, we discovered that in rats with POF, EBN had a specific impact on the uterine index, E2 level, and quantity of primordial, main, secondary, and sinus follicles. A tendency was observed for the number of primary, secondary, and sinus follicles to grow, and the rate of granulosa cell death decreased. Additionally, EBN tended to increase the FSHR protein's expression in the ovaries. These findings indicated that EBN products had both therapeutic and preventative effects on TG‐induced POF. Its mechanism of action might involve inhibition of follicular closure, control of hormones and receptors, and a decrease in ovarian granulosa cell death. This study comprehensively expounded on the improvement and mechanism of EBN on POF from phenotype and protein expression. However, it had not been explored from the aspect of gene regulation, which will be the focus of our follow‐up study.

As mentioned in the introduction, the pathogenesis of premature ovarian failure has not been fully explored, and the gene expression and signaling pathways related to POF should be studied deeply in the future. These related studies will help to further understand the causes of POF and provide a scientific basis for follow‐up treatment of POF. Furthermore, it is important to find and confirm more foods or drugs that have the function of improving premature ovarian failure, which can prevent and intervene in time.

## AUTHOR CONTRIBUTIONS

**Lin Jiang:** Data curation (lead); formal analysis (lead); writing – original draft (lead). **Xuncai Liu:** Investigation (lead); methodology (lead). **Fenghong Deng:** Validation (lead); visualization (lead); writing – review and editing (lead). **Yaxin Wang:** Project administration (lead); supervision (lead). **Qunyan Fan:** Conceptualization (lead); resources (lead).

## CONFLICT OF INTEREST STATEMENT

The authors declare no conflict of interest.

## Data Availability

The data that support the findings of this study are available from the corresponding author upon reasonable request.
